# Comparative Analysis of Mucosa-Associated and Luminal Gut Microbiota in Pediatric Ulcerative Colitis

**DOI:** 10.3390/ijms262110775

**Published:** 2025-11-05

**Authors:** Takeo Kondo, Sonoko Kondo, Haruyuki Nakayama-Imaohji, Ayano Tada, Nafisa Tabassum, Emmanuel Munyeshyaka, Kosuke Koyano, Shinji Nakamura, Takashi Kusaka, Tomomi Kuwahara

**Affiliations:** 1 Department of Pediatrics, Faculty of Medicine, Kagawa University, 1750-1 Ikenobe, Miki, Kita, Kagawa 761-0793, Japan; kondo.takeo@kagawa-u.ac.jp (T.K.); ijichi.sonoko@kagawa-u.ac.jp (S.K.); koyano.kosuke@kagawa-u.ac.jp (K.K.); nakamura.shinji@kagawa-u.ac.jp (S.N.); kusaka.takashi@kagawa-u.ac.jp (T.K.); 2 Department of Microbiology, Faculty of Medicine, Kagawa University, 1750-1 Ikenobe, Miki, Kita, Kagawa 761-0793, Japan; imaoji.haruyuki@kagawa-u.ac.jp (H.N.-I.); tada.ayano@kagawa-u.ac.jp (A.T.); s22d720@kagawa-u.ac.jp (N.T.); s22d730@kagawa-u.ac.jp (E.M.)

**Keywords:** pediatric ulcerative colitis, mucosa-associated microbes, 16S rRNA gene analysis, colonoscopy, intestinal lavage fluids

## Abstract

Inflammatory bowel diseases (IBD), including ulcerative colitis (UC) and Crohn’s disease, are chronic disorders relating to gut microbiota dysbiosis. Despite severe pancolitis being more prevalent in pediatric UC than in adults, alterations in the colon mucosa-associated microbiota (MAM) and their association with disease severity remain to be elucidated. The present study aimed to compare the gut microbiota in colon lavage fluids (CLFs) and fecal samples from 19 pediatric UC and 19 non-IBD patients. The community structure of MAM inferred by 16S metagenomic analysis was similar throughout the colon regardless of disease type. Bacterial compositions between MAM and feces were significantly different in non-IBD, while no difference was observed in pediatric UC, indicating a compromised mucous layer that could not sufficiently separate the MAM and luminal microbiota in UC. In pediatric UC, homogenous distribution of MAM was gradually disordered with increases in disease activity or mucosal inflammation, and bacterial groups of upper digestive tract or environmental origin were more abundant in MAM. Monitoring key bacterial markers in MAM, which include *Lactobacillus* and *Enterococcus* or *Faecalibacterium* and *Blautia* as increased or reduced members in pediatric UC, respectively, might be useful for evaluation of patient prognosis.

## 1. Introduction

Inflammatory bowel diseases (IBD), which include ulcerative colitis (UC) and Crohn’s disease (CD), are chronic inflammatory conditions of the gastrointestinal tract with unknown etiology. Complex factors such as disruption of gut mucosal barrier function, dysbiosis of gut microbiota, excessive gut immune response to resident microbes, and Westernized lifestyle are considered to increase the risk of IBD [1]. Genetic background also seems to be involved in IBD pathogenesis. Genome-wide association studies have revealed 163 distinct genetic loci associated with IBD; some of these loci are related to mucosal barrier function and host innate immunity to gut microbiota [2].

The prevalence of childhood-onset IBD has increased rapidly over the last few decades [3,4,5]. Pediatric IBD patients show several clinical features that differ from those in adults. Among pediatric UC patients, pancolitis is more common, and disease severity is higher than in adults [6]. In addition, the control of disease activity is often more difficult due to the longer duration of gut inflammation [7].

In recent years, metagenomics and metabolomics have brought novel insights regarding the relationships of the human microbiome with various diseases. There is growing evidence showing that the reduction in biodiversity in gut microbiota is prolonged in IBD [8]. This reduction leads to gut mucosal inflammation due to insufficient supply of health-promoting metabolites from the microbiota, colonization of potentially pathogenic microorganisms, and thinning of the intestinal mucus layer, allowing the gut microbes to directly adhere to epithelial cells [9]. This condition is called “dysbiosis,” which is considered to play an important role in IBD pathophysiology. Dysbiosis induces alteration of microbiota composition. Previous studies have also shown that the gut microbial communities of patients with IBD are characterized by reduced diversity as well as concomitant decreases and increases in the phyla Firmicutes and Proteobacteria, respectively [10,11,12].

The gut microbiota consist of luminal and mucosa-associated microbiota (MAM). Compared with luminal microbiota, MAM have been reported to play a more important role in the maintenance of intestinal homeostasis and host immunity in patients with IBD [13]. Some authors have reported that compared with healthy controls, patients with IBD have higher amounts of bacteria attaching to their gut epithelial surfaces, even in the non-inflamed mucosa [14,15].

Studies using biopsy samples and brush scrapings collected by colonoscopy have been conducted in adults; these studies found that the composition of intestinal MAM differs greatly from those of fecal samples [16]. Various reports have shown differences in the diversity and composition of the microbiota between the feces and biopsy samples from healthy adults [17,18]. Similar studies have been conducted in IBD. Chen reported differences in microbiota composition in stool and intestinal MAM between healthy subjects and UC patients [19]. Another study compared the microbiome in stool and colonic biopsy samples from healthy adults and IBD patients, reporting that the microbiota in fecal samples from healthy subjects and IBD patients were similar, but the differences in colonic MAM between the groups were evident [20].

Most of the previous studies on intestinal MAM used biopsy samples. However, the procedure is invasive, particularly for children, and biopsy samples reflect the intestinal MAM at limited mucosal regions. Although fecal samples are non-invasive, they may not accurately represent the microbiota at the mucosal interface where host-microbe interactions occur. Less invasive sampling, such as brush scrapings or lavage fluid collection, has been employed to examine the differences in intestinal MAM between healthy adults and patients with IBD [21]. At present, only limited information is available for intestinal MAM in children, in which biopsy samples [22,23,24] or intestinal lavage fluid [25] were examined in pediatric UC. However, the latter study compared the duodenal MAM between pediatric UC patients and healthy controls. There have been no reports so far on studies that examined the colon lavage fluids in pediatric UC.

In the present study, we performed a 16S rRNA gene amplicon sequencing analysis of the MAM using endoscopic lavage fluids collected throughout the colon (ascending colon, transverse colon, sigmoid colon, and rectum) in pediatric UC patients. These microbiome data were compared with those of fecal samples. We also aimed to identify the microbes that correlate with the clinical activity of UC or the serological inflammatory markers.

## 2. Results

### 2.1. Cohort and Sample Characteristics

The demographic and clinical characteristics of the patients are summarized in Table 1. A total of 38 children, aged 3–19 years (11.4 ± 3.6), were enrolled, and 140 colonic lavage fluid (CLF) samples were collected from different sites of the large intestine (ascending colon, transverse colon, sigmoid colon, and rectum). Twenty-three fecal samples were also collected. UC patients were treated according to the guidelines [26,27]. Three UC and one non-IBD patient had received antibiotics within 4 weeks before sampling.

### 2.2. Association of Host Factors with Gut Microbial Taxonomic Variation in UC and Non-IBD

**Figure 1:**
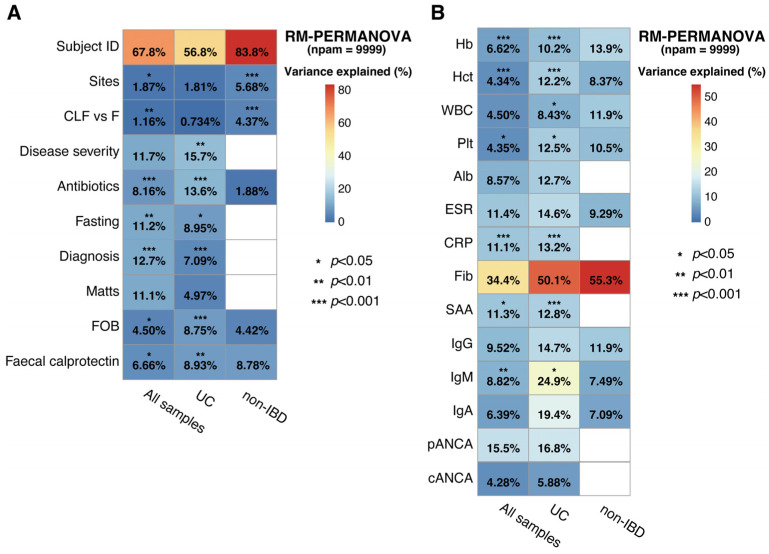
Association of gut microbial community structures with various clinical and laboratory characteristics in pediatric UC patients. (**A**) Contribution of the sample profiles, sampling sites, sample types, disease severity (PUCAI), disease activity (remission or active), mucosal integrity (Matts), fecal occult blood, fecal calprotectin level, and treatments to microbial taxonomic variations in pediatric UC or non-IBD patients. The measurement of each variable is summarized in Table S1. (**B**) Microbial taxonomic variation plotted against blood and serological analysis data of the patients. The measurement of each variable is summarized in Table S1. The statistical difference was evaluated by repeated measures PERMANOVA (RM-PERMANOVA) with 9999 permutations. Asterisks indicate the level of statistical significance (* *p* < 0.05, ** p < 0.01, *** p < 0.001). The percentage values within the heatmap represent the proportion of microbial community variance explained by each variable. Abbreviations: Alb, albumin; cANCA, cytoplasmic antineutrophil cytoplasmic antibodies; CRP, C-reactive protein; ESR, erythrocyte sedimentation rate; F, feces; Fib, fibrinogen; FOB, fecal occult blood; Hb, hemoglobin; Hct, hematocrit; IBD, inflammatory bowel disease; pANCA, peripheral antineutrophil cytoplasmic antibodies; Plt, platelet count; PUCAI, Pediatric Ulcerative Colitis Activity Index; SAA, serum amyloid A protein; UC, ulcerative colitis; WBC, white blood cell count.

We firstly conducted the association analysis using microbial abundance data and sample metadata to identify the factors that impact the gut microbiome in pediatric UC (Figure 1). As seen in “Subject ID”, which specified the patient, inter-individual difference explained >50% of the variation in the gut microbiome in both UC and non-IBD (Figure 1A). Inter-individual difference was more evident in non-IBD than UC, which contributed to 83.8% and 56.8% variation in the gut microbial community structure, respectively. The community structure in the MAM was similar throughout the colon in both UC and non-IBD. Of note, microbiota composition in CLF and feces was similar in UC (see CLF vs. F in Figure 1A). On the other hand, significant difference was detected in both α-diversity (number of observed operational taxonomic units [OTUs]) and β-diversity among the microbiota in CLFs and feces in non-IBDs although the microbiota compositions in CLFs were similar (Figure S1). The α-diversity of MAM was unexpectedly higher than that of fecal microbiota in non-IBD patients. The association analysis showed a significant association of disease severity and antibiotic treatment with the variation in the gut microbiome of UC patients, which explained more than 10% of the variation in gut microbial taxonomy in UC.

Among the blood and serological examinations, serum levels of acute phase proteins (fibrinogen, C-reactive protein [CRP], and serum amyloid A [SAA]) showed a strong association with microbiome composition in both CLF and feces of UC, with fibrinogen accounting for more than 50% of the variation in microbial taxonomy (Figure 1B). The levels of immunoglobulins, especially IgM, showed significant association with the gut microbial taxonomic variation in UC.

### 2.3. Association of the Specific Microbial Taxa with Clinical Metadata

**Figure 2:**
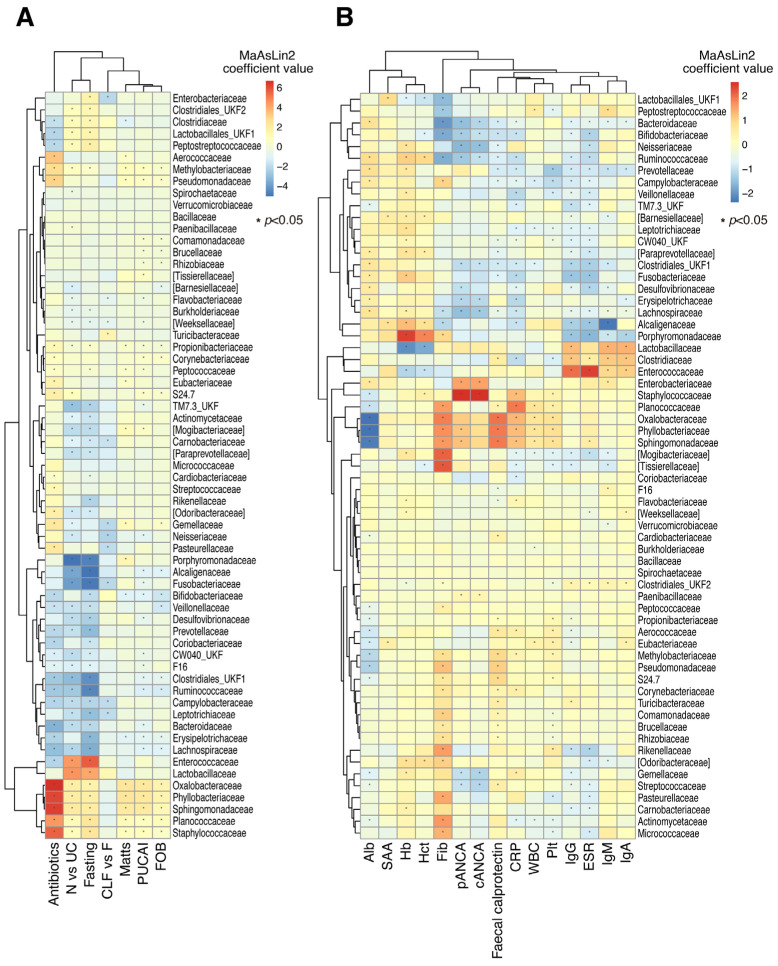
Association of bacterial taxa (family level) detected in CLF or feces with clinical metadata. The association was evaluated by linear model coefficients (MaAsLin2). The association strength is shown by the coefficient value with color gradation (red, positive; blue, negative). An asterisk (*) indicates a statistically significant association, which is defined as a False Discovery Rate (FDR)-adjusted *p*-value < 0.05. (**A**) Association of the bacterial taxa with disease type (non-IBD vs. UC), sampling sites, sample types, disease severity (PUCAI), disease activity (remission or active), mucosal integrity (Matts), FOB, fecal calprotectin level, or treatment in the pediatric patients. (**B**) Association of bacterial taxa with blood and serological analysis data of the pediatric patients. Details of the clinical data are summarized in Table S1. Abbreviations: Alb, albumin; cANCA, cytoplasmic antineutrophil cytoplasmic antibodies; CLF, colon lavage fluids; CRP, C-reactive protein; ESR, erythrocyte sedimentation rate; F, feces; Fib, fibrinogen; FOB, fecal occult blood; Hb, hemoglobin; Hct, hematocrit; IBD, inflammatory bowel disease; pANCA, peripheral antineutrophil cytoplasmic antibodies; Plt, platelet count; PUCAI, Pediatric Ulcerative Colitis Activity Index; SAA, serum amyloid A protein; UC, ulcerative colitis; WBC, white blood cell count; UKF, unknown family.

To identify the specific association of microbial taxa (family level) with clinical data, we conducted an association analysis by linear model coefficients (MaAsLin2). Figure 2A shows the specific microbial groups strongly associated with disease type (non-IBD vs. UC), fasting, and antibiotic treatment. In UC, the relative abundances of Enterococcaceae and Lactobacillaceae were higher, while those of Porphyromonadaceae, Alcaligenaceae, and Fusobacteriaceae were lower than in non-IBD. Antibiotic treatment correlated with the increase in the relative abundances of Oxalobacteraceae, Phyllobacteriaceae, Sphingomonadaceae, Planococcaceae, and Staphylococcaceae. Of these, the first three families were negatively correlated with serum albumin and positively correlated with fecal calprotectin levels (Figure 2B). No microbial taxa were significantly associated with sample type (CLF vs. F), mucosal inflammation (Matts), disease severity (PUCAI), or fecal occult blood level. Analysis at the genus level showed that the abundances of *Lactobacillus* and *Enterococcus* were higher, while those of *Blautia*, *Faecalibacterium*, and *Parabacteroides* were lower in UC when compared with non-IBD gut microbiome (Figure S2). Antibiotic treatment was associated with increased abundances of environmental bacteria such as *Ralstonia* and *Phyllobacterium* as well as *Staphylococcus*. While environmental bacteria such as *Ralstonia*, *Phyllobacterium*, and *Sphingomonas* were negatively correlated with serum albumin levels, they showed significant associations with inflammatory markers such as CRP and fecal calprotectin levels. *Faecalibacterium* and *Parabacteroides* showed positive correlations with hemoglobin (Hb) and hematocrit (Hct) levels. Diverse microbial genera were associated with serum fibrinogen level. Interestingly, *Lactobacillus* and *Enterococcus* showed significant associations with all classes of immunoglobulins (IgG, IgM, and IgA). The levels of autoantibodies pANCA and cANCA were significantly associated with the relative abundances of *Staphylococcus* and unassigned genus within Enterobacteriaceae in gut microbiome (Figure S2).

### 2.4. Homogenous Distribution of Microbiota in MAM Along the Colon

**Figure 3:**
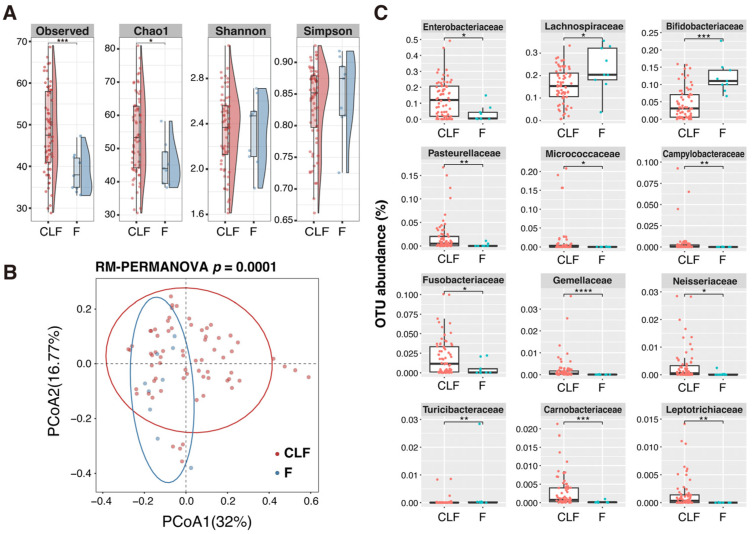
Difference in microbiota composition between CLF and feces in non-IBD patients. (**A**) Comparison of α-diversity between the gut microbiomes in CLF and F samples. The *y*-axis represents the values for various alpha-diversity indices (observed number of genus, Chao1, Shannon, and Simpson). Asterisks indicate significant differences as determined by a linear mixed-effects model (* *p* < 0.05, *** *p* < 0.001). (**B**) PCoA plot based on Bray–Curtis dissimilarity, showing that the microbial communities of CLF and F form statistically distinct clusters (*p* = 0.0001; RM-PERMANOVA, 9999 permutations). The circles indicate 95% confidence ranges for each sample, respectively. (**C**) Relative abundance of bacterial families that were significantly different between CLF and F samples. The *y*-axis represents the OTU abundance (%). Asterisks indicate significant differences as determined by the Wilcoxon rank-sum test (* *p* < 0.05, ** *p* < 0.01, *** *p* < 0.001, **** *p* < 0.0001). Abbreviations: CLF, colon lavage fluid; F, feces; PCoA, principal coordinate analysis; RM-PERMANOVA, repeated measures PERMANOVA; OTU, operational taxonomic unit.

We investigated the differences between the microbial community structures of MAM and fecal microbiota. Significant differences were observed in α- and β-diversities between MAM and fecal microbiota in non-IBD pediatric patients (Figure 3A,B). The microbiota in CLF contained a larger number of OTUs (family level) than did feces. The abundances of Lachnospiraceae and Bifidobacteriaceae were significantly higher in feces, whereas those of Enterobacteriaceae and Campylobacteriaceae were significantly higher in CLF (Figure 3C). Oropharyngeal bacteria such as Pasteurellaceae, Fusobacteriaceae, Gemellaceae, Neisseriaceae, and Leptotrichiaceae were also significantly more enriched in CLF than in feces in non-IBD pediatric patients.

**Figure 4:**
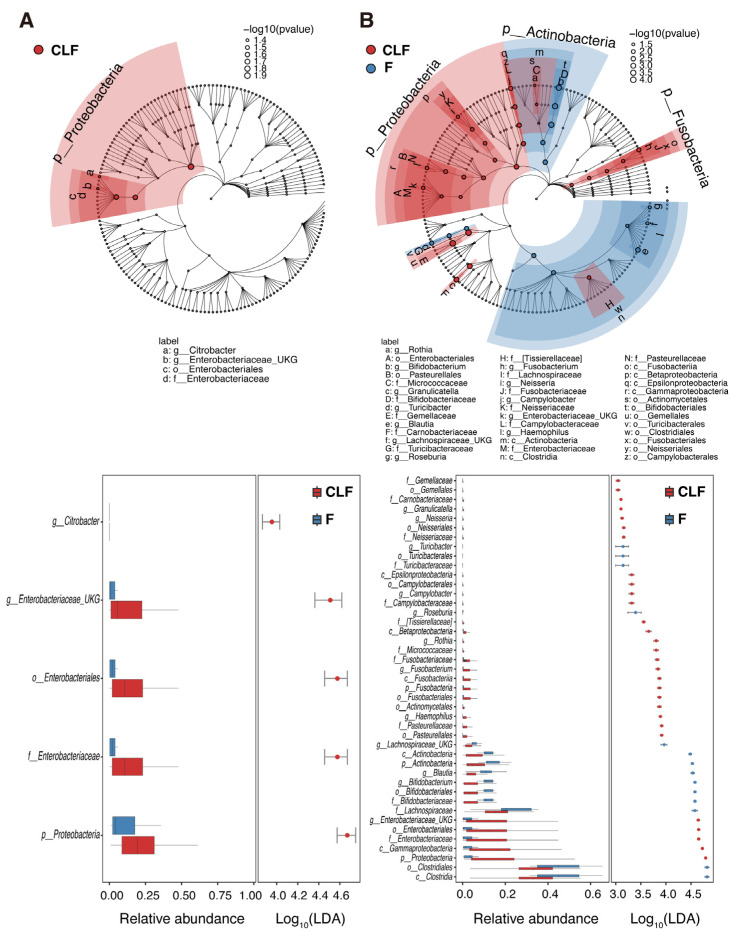
Comparison of the microbial community structures in CLF versus feces at various taxonomic levels. Cladograms were built by LEfSe analysis utilizing the relative abundance data of bacterial taxa detected in CLF and F samples from pediatric UC (**A**) and non-IBD patients. (**B**) The bar plots below each cladogram show the linear discriminant analysis (LDA) scores and relative abundances of the taxa differentially enriched in each group. Prefixes (*p*, *o*, *c*, *f*, and *g*) indicate taxonomic level (phylum, class, order, family, and genus, respectively). Bacterial taxa enriched in either of the two sample types (CLF or F) are indicated by color (LDA score > 3.0, the taxa enriched in CLF shown in red while those in F are shown in blue). Abbreviations: CLF, colon lavage fluids; F, feces; IBD, inflammatory bowel disease; LDA, linear discriminant analysis; LEfSe, linear discriminant analysis effect size; UC, ulcerative colitis.

On the other hand, no significant difference in microbiota diversity between CLF and feces was observed in pediatric UC patients (Figure S3). Enterobacteriaceae was the only bacterial group that was significantly different in abundance between MAM and fecal microbiota. Consistent with the non-IBD findings described above, LEfSe algorithm analysis showed that genera within Enterobacteriaceae made up the only bacterial group that was higher in MAM than in fecal microbiota in pediatric UC patients (Figure 4A). In the case of non-IBD patients, the relative abundance of various bacterial groups was significantly different between MAM and fecal microbiota, where Clostridia, Lachnospiraceae, Bifidobacteriaceae, *Blautia*, and *Roseburia* were more abundant in feces, while Enterobacteriaceae, Campylobacteriaceae, and oral/oropharyngeal bacteria such as Neisseriaceae, Pasteurellaceae, Gemellaceae, and Fusobacteriaceae were more abundant in MAM (Figure 4B). These results indicate that compartmentalized distribution of gut microbiota along the mucosa-luminal axis was disrupted in the pediatric UC patients.

### 2.5. Microbial Community Structure of MAM and Feces in Pediatric UC and Non-IBD Patients

**Figure 5:**
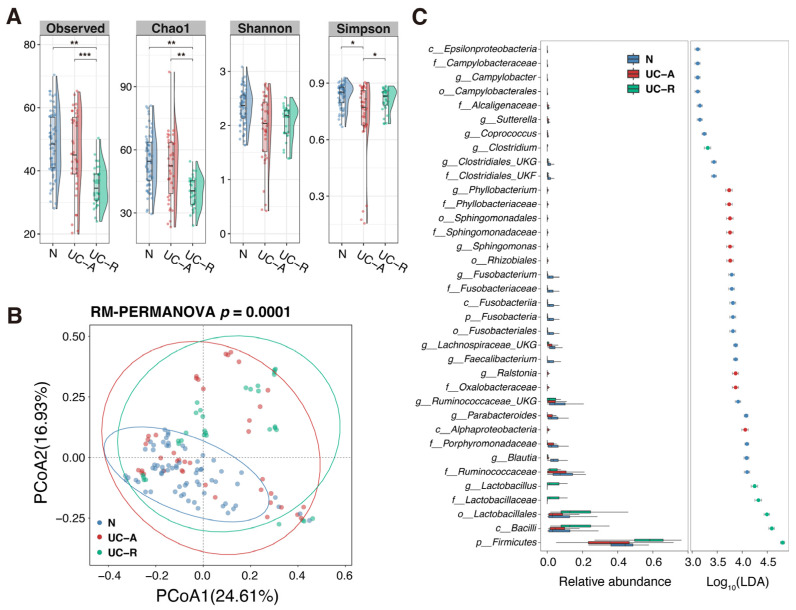
Difference between microbial community structures in CLF between pediatric UC and non-IBD patients. (**A**) Comparison of α-diversity indices of the microbiota in CLF among the non-IBD (N), active UC (UC-A), and remission UC (UC-R) groups. The *y*-axis represents the values for four different indices (observed number of genus, Chao1, Shannon, and Simpson). Asterisks indicate significant differences between groups as determined by a linear mixed-effects model (* *p* < 0.05, ** *p* < 0.01, *** *p* < 0.001). (**B**) PCoA plot based on Bray–Curtis dissimilarity, showing distinct clustering of the microbial communities in CLF among the N, UC-A, and UC-R groups. The circles indicate 95% confidence range for each group, respectively. The overall difference was statistically significant (*p* = 0.0001; RM-PERMANOVA, 9999 permutations). (**C**) LEfSe analysis identifying taxa within the microbiota in CLF that are differentially abundant among the N, UC-A, and UC-R groups. The left plot shows the relative abundance of each taxon, and the right plot shows its corresponding linear discriminant analysis (LDA) score. Abbreviations: LDA, linear discriminant analysis; N, non-IBD (non-inflammatory bowel disease); UC-A, active ulcerative colitis; UC-R, remission ulcerative colitis; PCoA, principal coordinate analysis; RM-PERMANOVA, repeated measures PERMANOVA.

The microbial community structure of MAM was homogenous across the tested colon sites in both UC and non-IBD patients (Figure S1). We next addressed the difference in MAM between the patient groups. As shown in Figure 5A, the α-diversity indices (Observed number of genus, Chao1, and Simpson) were significantly lower in the active UC (UC-A) and remission UC (UC-R) groups compared to the non-IBD (N) group. PCoA showed significantly different distributions between UC and non-IBD, where the community structure of MAM was more variable within the samples of the UC groups (between UC-A and UC-R) than within non-IBD (Figure 5B). LEfSe analysis identified the differentially enriched bacterial groups in the MAM between UC and non-IBD patients (Figure 5C). The abundance of the bacterial groups within Ruminococcaceae, *Blautia*, Porphyromonadaceae, *Parabacteroides*, *Faecalibacterium*, Lachnospiraceae, and *Fusobacterium* was higher in MAM of non-IBD. In contrast, groups belonging to Lactobacillales and *Lactobacillus* were higher in the MAM of the UC-R patients, while those belonging to *Ralstonia*, *Sphingomonas*, and *Phyllobacterium* were higher in the MAM of the UC-A patients. The same analysis was conducted with fecal microbiota (Figure S4). In contrast to the results in MAM, no significant differences were observed between the groups in either α-diversity or β-diversity of the fecal microbiota. In addition, no bacterial group enrichment was detected in UC fecal microbiota, while typical fecal microbes in healthy subjects, such as Lachnospiraceae, *Blautia*, and *Parabacteroides*, were enriched in non-IBD patients. These results strongly suggest that MAM, rather than feces, is a more sensitive sample type for reflecting the mucosal dysbiosis associated with UC.

### 2.6. Alteration in Community Structure of MAM Depending on UC Disease Activity

**Figure 6:**
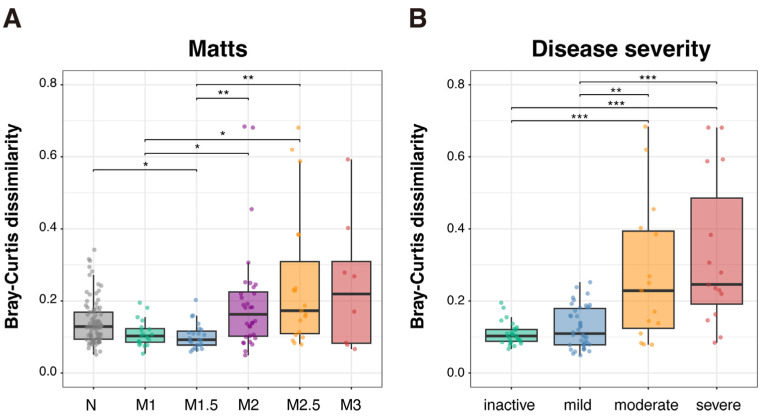
Regional dissimilarity in community structure of MAM increased depending on mucosal inflammation and disease severity. Bray–Curtis distance among the four sampling sites (ascending, transverse and sigmoid colon and rectum) was calculated for every individual, using genus-level microbiome abundance data. (**A**) Relationship between mucosal inflammation and regional difference in MAM composition. The *x*-axis represents the non-IBD group (N) and UC patients categorized by their mean Matts score calculated from the four colonic sampling sites (M1, M1.5, M2, M2.5, and M3), reflecting the overall degree of mucosal inflammation. (**B**) Relationship between disease severity and regional difference in MAM composition. The *x*-axis represents the disease activity categories based on the PUCAI score (inactive, mild, moderate, and severe). The data of Matts scores and disease severity (PUCAI) are summarized in Table S1. Significant differences, as determined by the Wilcoxon rank-sum test, are shown by * (*p* < 0.05), ** (*p* < 0.01), or *** (*p* < 0.001). Abbreviations: MAM, mucosa-associated microbiome; non-IBD, non-inflammatory bowel disease; PUCAI, Pediatric Ulcerative Colitis Activity Index.

As described above, the community structure of MAM was homogenous along the colon in both UC and non-IBD, although the microbiome patterns were different between the groups. We examined whether microbial abundance in MAM differs according to mucosal inflammation or disease severity of pediatric UC patients. As shown in Figure 6, intra-individual Bray–Curtis distance increased with the degree of mucosal inflammation and disease severity. Spearman’s rank correlation analysis confirmed that alteration in MAM within each patient significantly correlated with both the mean Matts score (*rho* = 0.399, *p* < 0.001) and disease severity (PUCAI) (*rho* = 0.467, *p* < 0.001). These results indicate that mucosal inflammation and disease severity are associated with the regional heterogeneity of MAM in UC.

### 2.7. Profiling of MAM Community Structure According to UC Disease Severity

**Figure 7:**
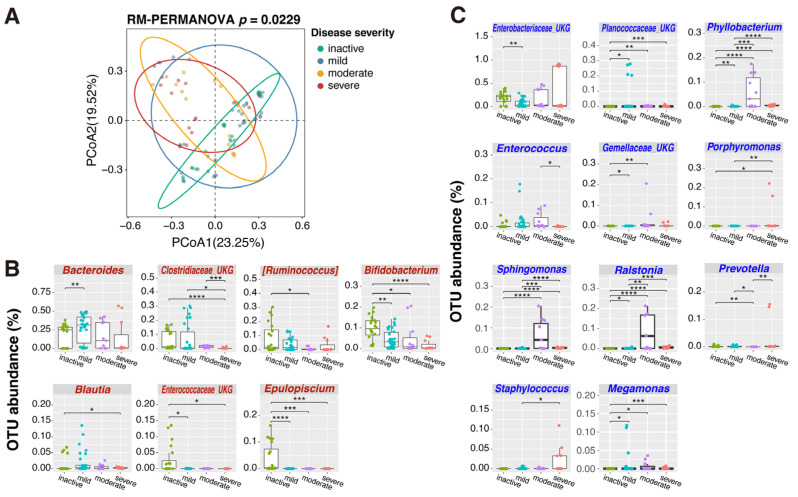
Association of community structure of MAM with UC disease activity. The patients were classified into four disease activities according to PUCAI (Table S1). (**A**) PCoA plot of microbiome in MAM according to disease activity. The overall difference was statistically significant (*p* = 0.0229; RM-PERMANOVA, 9999 permutations). The circles indicate 95% confidece range for each category, respectively. (**B**) Bacterial genera whose relative abundances in MAM are negatively correlated with disease severity. (**C**) Bacterial genera whose relative abundances in MAM are positively correlated with disease severity. In panels (**B**,**C**), only the bacterial groups with relatively high abundance (>0.1%) in any of the samples are shown. The *y*-axis represents the OTU abundance (%). Significant differences as determined by the Wilcoxon rank-sum test are shown by * (*p* < 0.05), ** (*p* < 0.01), *** (*p* < 0.001), or **** (*p* < 0.0001). Abbreviations: MAM, mucosa-associated microbiome; OTU, operational taxonomic unit; PCoA, principal coordinate analysis; PUCAI, Pediatric Ulcerative Colitis Activity Index; RM-PERMANOVA, repeated measures PERMANOVA; UC, ulcerative colitis.

We analyzed the association of the microbial community structure of MAM with UC disease activity. Samples were classified into four groups (inactive, mild, moderate, and severe) depending on PUCAI. PCoA analysis of the microbiome data showed that the community structure of MAM changed with exacerbation of the disease (Figure 7A). Among the genera of relatively high abundance (>0.1% in either of the samples), *Bifidobacterium*, *Ruminococcus*, *Blautia*, *Epulopiscium*, and unassigned OTU within Clostridiaceae and Enterococcaceae decreased in the MAM with disease severity (Figure 7B). However, *Bacteroides*, *Phyllobacterium*, *Enterococcus*, *Porphyromonas*, *Sphingomonas*, *Ralstonia*, *Prevotella*, *Staphylococcus*, *Megamonas*, and unassigned OTU within Enterobacteriaceae, Planococcaceae, and Gemellaceae were higher in the MAM of the UC patients with active disease (Figure 7C). DESeq2 analysis was conducted to detect which OTUs in MAM (including minor populations) were differentially enriched (>4 fold change) depending on disease severity (Figure S5). Many bacterial groups had different abundances when MAM was compared between inactive and active UC patients. Abundances of *Lachnospira*, *Epulopiscium*, and OTU within Enterococcaceae were lower, while those of diverse bacterial groups such as *Phascolarctobacterium*, *Edwardsiella*, *Pediococcus*, *Planococcus*, *Sphingomonas*, *Ralstonia*, *Phyllobacterium*, and unassigned genera within Barnesiellaceae were higher in MAM from active UC. In comparisons between the patient groups, the number of differentially enriched bacterial groups in MAM decreased as disease activity progressed. *Edwardsiella*, *Staphylococcus*, *Aggregatibacter*, and the bacterial groups in Coriobacteriaceae and Enterobacteriaceae were enriched in MAM of the patients with severe disease activity. Interestingly, the abundance of probiotic *Lactobacillus* and unassigned OTU within Bifidobacteriaceae was higher in mild and moderate disease activity, respectively, when compared with inactive stage disease.

### 2.8. Profiling of MAM Community Structure According to Mucosal Inflammation

**Figure 8:**
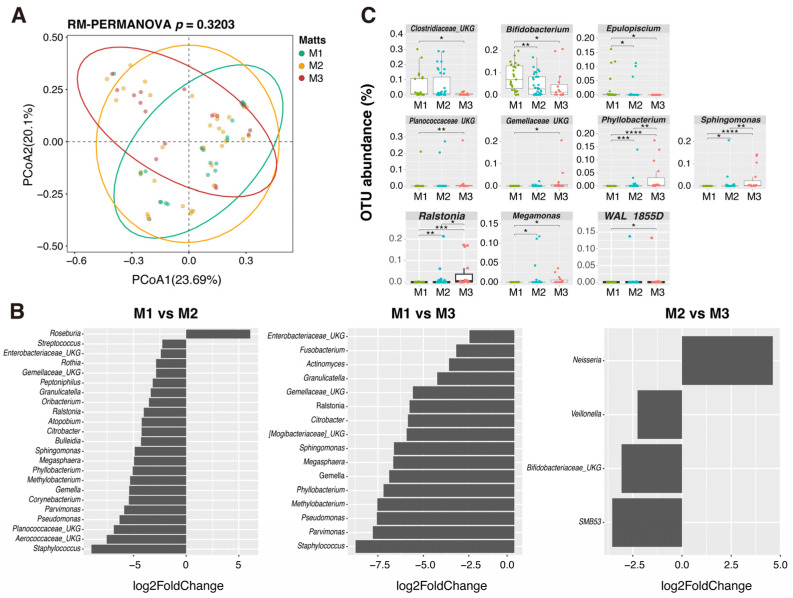
Microbial community structures of MAM and their relationship with mucosal inflammation in pediatric UC. (**A**) PCoA plot of microbiome in MAM categorized by Matts score. The overall difference among groups was not statistically significant (*p* = 0.3203; RM-PERMANOVA, 9999 permutations). The circles indicate 95% confidence range for each category, respectively. (**B**) Bacterial genera identified by DESeq2 analysis as being differentially enriched among different degrees of mucosal inflammation (M1 vs. M2; M1 vs. M3; M2 vs. M3). The bar plots show the log2 fold change in abundance. A positive value indicates enrichment in the former (e.g., ‘M1’ for the ‘M1 vs. M2’ comparison), while a negative value indicates enrichment in the latter. Only genera with an FDR-adjusted *p*-value < 0.05 and a fold change greater than 4 are shown. (**C**) Relative abundance of specific bacterial genera that changed according to the Matts score grade. The y-axis represents the OTU abundance (%). M1, M2, and M3 correspond to the Matts endoscopic score grades for mucosal inflammation: M1,normal mucosa; M2, mild inflammation; M3, moderate/severe inflammation. Significant differences examined by Wilcoxon rank-sum test in panel (**C**) are shown by * (*p* < 0.05), ** (*p* < 0.01), *** (*p* < 0.001) or **** (*p* < 0.0001). Abbreviations: MAM, mucosa-associated microbiome; OTU, operational taxonomic unit; PCoA, principal coordinate analysis; RM-PERMANOVA, repeated measures PERMANOVA; UC, ulcerative colitis; UKG, unknown genus.

Mucosal inflammation of the sampling site was endoscopically evaluated by Matts scoring (Table S1). To investigate the impact of mucosal inflammation on the microbial community structure of MAM in pediatric UC, PCoA was conducted using the relative abundances of genus-level OTUs. However, the overall microbial community structure (β-diversity) of MAM was not significantly altered by the degree of mucosal inflammation as evaluated by the Matts score (Figure 8A). DESeq2 analysis detected no OTU enrichment in MAM on normal mucosa (M1 vs. M2 or M3) except for *Roseburia* (Figure 8B. On the other hand, the community structure of MAM on the inflamed mucosa (M2 vs. M3) was similar and enriched in bacterial groups that mainly colonize the oral cavity or upper digestive tract (e.g., *Parvimonas* and *Gemella*) and that have environmental origins (e.g., *Pseudomonas*, *Phyllobacterium*, and *Ralstonia*) compared with normal mucosa (M1). It was noteworthy that *Staphylococcus* was the most abundant group in MAM on inflamed mucosa (M2 and M3). Consistent with the results shown in Figure S5, the unknown genera within Bifidobacteriaceae retained their abundances in MAM on inflamed mucosa. Among the bacterial groups of high abundance, the OTUs within Clostridiaceae, *Bifidobacterium*, and *Epulopiscium* decreased in abundance, while *Phyllobacterium*, *Sphingomonas*, *Megamonas*, *Ralstonia,* and the unassigned OTUs within Planococcaceae and Gemellaceae gradually increased depending on Matt’s grade (Figure 8C).

## 3. Discussion

Although intestinal MAMs have been observed to be involved in the pathophysiology of UC, many of the previous studies focusing on MAM used mucosal biopsy in adults as well as pediatric patients [28,29,30]. In this study, we employed CLFs and paired fecal samples to determine specific alterations in MAM according to mucosal inflammation or disease activity. We collected CLFs using colonoscopy via a sterile tube and carried out high-throughput 16S rRNA gene metagenomic analysis. In order to evaluate the microbiota residing proximal to the mucous layer, we aspirated the CLFs immediately after vigorously washing the mucosal surfaces using the waterjet function of the endoscope. This method has advantages for safe sampling from a wide mucosal area along the colon because biopsy at many sites is invasive, especially for children. In addition, the lavage contains a sufficient number of microbial cells, which minimizes the effect of environmental contamination on PCR-based analysis and allows more efficient isolation of the microbes of interest. To our knowledge, this is the first report to conduct a comparative analysis of microbiota between CLFs and paired samples of feces from pediatric UC patients.

The results in our study using CLF are largely consistent with many previous reports that used biopsy samples [16,17,18,20,21,22,23,24]. For example, studies on biopsy samples from both adult and pediatric UC patients have reported an increase in the phylum Proteobacteria (particularly the family Enterobacteriaceae) and a decrease in the phylum Firmicutes, including butyrate-producing bacteria such as *Faecalibacterium* and *Blautia*, compared to healthy controls. Our analysis of MAM in CLF also showed that Enterobacteriaceae tended to be more abundant in UC patients, while *Blautia* and *Faecalibacterium* were enriched in the non-IBD group (Figure 5C), which aligns well with the trends in previous reports. This concordance suggests that the less-invasive CLF sampling method can effectively capture the pathophysiological changes in MAM.

Consistent with other reports [31,32,33], substantial inter-individual differences in gut microbiome were observed among the patients (Figure 1). However, these differences accounted for less variation in the UC than in the non-IBD microbiomes. In addition, compared with UC, the microbiome variations in non-IBD correlated more with sampling sites (A, T, S, R, or F) and sample types (CLF or feces). The microbiome composition (β-diversity) of MAM in UC was homogenous along the colon and similar to that in feces. However, in the cases of non-IBD, the microbiomes in MAM tended to be different from those in feces (Figure 3). Antibiotics are a well-known factor that affects the gut microbiome. Although our study cohort contained five patients (four UC and one non-IBD patient) with a history of antibiotic treatment within four weeks before sampling, the same trends were observed when the data from these patients were excluded from the analysis (Figure S7). These results suggest that compartmentalized distribution of the microbiome along the mucosa-luminal axis is disrupted in UC due to the compromised mucous layer function even in the remission stage. The homogenous distribution of intestinal MAM might account for the higher prevalence of severe pancolitis in pediatric UC than in adult cases [34,35]. Age-dependent alteration in MAM along the intestine should be investigated to address the precise role of the MAM on the pathophysiology in UC.

Among the blood analysis data, the serum levels of all classes of immunoglobulins (IgA, IgG, and IgM) were associated with the variation in the MAM, and the degree of correlation was more evident in UC than in non-IBD. MaAsLin2 analysis showed that abundances of Enterococcaceae and Lactobacillaceae were strongly associated with these antibody levels, indicating that UC patients developed humoral immune responses to these bacterial groups (Figure 2). These findings were consistent with the report by Bourgonje et al., in which *Streptococcus*, *Lactobacillus*, *Lactococcus*, *Enterococcus*, *Veillonella*, and Enterobacteriaceae were enriched among IgG-coated bacteria in adult UC [36]. In UC, levels of the autoantibodies pANCA and cANCA correlated with the relative abundance of *Staphylococcus* and Enterobacteriaceae (Figure 2). This association was probably due to the strong inflammatory response evoked by these bacteria. Luo et al. reported that systemic translocation of *Staphylococcus* was associated with germinal center B cell activation and production of autoantibodies to nuclear antigen (ANA) and double strand DNA [37]. They also showed a higher plasma LPS level in HIV-positive patients with high ANA than with low ANA levels. It was also reported that *E. coli*-derived caseinolytic protease B induces autoantibody formation [38]. Overall, the levels of serum inflammatory markers and levels of immunoglobulin were more correlated with the MAM than fecal microbiota (Figure 1), suggesting that the alteration in MAM reflects the host humoral immune response to gut microbiota.

Unexpectedly, the microbiota within CLFs from non-IBD pediatric patients showed higher microbial diversity compared with feces, and the bacterial families usually residing in oropharynx or upper intestines, such as Enterobacteriaceae, Pasteurellaceae, Fusobacteriaceae, and Neisseriaceae, were significantly more abundant in the MAM. This may reflect the differences in nutrient availability (e.g., mucin vs. dietary fiber) and interaction with the host immune system between the colonic mucosa and the luminal part of the colon. This could allow a diverse bacteria to adapt to the oral cavity or upper intestine, where the bacteria reside in proximity to epithelial cells, to colonize the colonic mucous layer. On the other hand, Bifidobacteriaceae and Lachnospiraceae, representative luminal members of gut microbiota, were more abundant in feces (Figure 3 and Figure 4). Microbiota of oral origin are known to increase in the intestines of IBD patients [39]. Schirmer et al. reported that *Veillonella dispar*, *Veillonella parvula*, *Aggregatibacter segnis*, *Haemophilus parainfluenzae*, *Campylobacter* sp., Lachnospiraceae, *Megasphaera* sp. increased in the intestine of treatment-naïve pediatric UC patients with the disease course [40]. Somineni et al. reported that an increase in oral bacteria before treatment was associated with aggravation of UC [41]. Moreover, Atarashi et al. reported that oral microbiota were significantly more abundant in the fecal samples from UC patients compared with healthy controls. In addition, they showed that *Klebsiella* sp. isolated from the salivary microbiota are strong inducers of Th1 cells when they colonize in the gut [42].

Originally, normal intestinal microbiota resist colonization of foreign microorganisms, which suppresses the colonization and proliferation of pathogenic bacteria. It has been reported that the intestinal tracts of IBD patients are more likely to be colonized by oral microbiota, resulting from attenuated colonization resistance [43,44]. In addition, an increase in oral bacteria migrating to the intestinal tract has been observed in those patients. In IBD patients, genetic factors, treatments, and intestinal inflammation may increase the abundance of swallowed oral bacteria that reach the lower intestine [45,46,47]. However, our data showed that the relative abundances of these bacteria in CLF from non-IBD were higher than those from feces (Figure 5), indicating that they normally reside in proximity to the mucous layer due to their adhesive properties and/or relative tolerance to reactive oxygen species. On the other hand, in MAM from the pediatric UC patients, we detected only enrichment in Enterobacteriaceae. These results indicate that the compromised mucous layer in UC had lost the ability to separate MAM and luminal microbiota, and also that Enterobacteriaceae is more capable of surviving in inflamed mucosa.

In agreement with other reports [48], mucosal inflammation or disease activity correlated with the alteration in MAM (Figure 6). As Matts score (mucosal inflammation) or PUCAI (disease activity) increased, the Bray–Curtis dissimilarity in MAM among the sites (A, T, S and R) became larger, suggesting that the UC disease severity promotes the intra-individual regional variations in MAM by selective pressures (e.g., oxidative damage or clearance by the immune system). We detected the differentially enriched bacterial genera at each disease condition determined by Matts score or PUCAI (Figure 7 and Figure 8). In terms of disease activity, the relative abundances of representative luminal members such as *Bacteroides*, *Ruminococcus*, *Bifidobacterium*, and *Blautia* decreased with disease severity, while those of skin and environmental bacteria (*Phyllobacterium*, *Sphingomonas*, *Ralstonia*, Planococcaceae, and *Staphylococcus*) and oral and upper intestinal tract origin (Gemellaceae_UKG, *Porphyromonas*, and *Prevotella*) increased. A similar trend was observed regarding the Matts score. Among the genera belonging to Enterococcaceae (*Enterococcus* and unassigned genus), some increased and others decreased. This difference is probably due to variations in proinflammatory potential among *Enterococcus* species, as described previously [49]. A larger number of differentially enriched genera (>4-fold change) was detected in the analysis based on disease activity compared with Matts score (Figure 8 and Figure S5), again suggesting that gut physiological dysfunction, compared with local mucosal inflammation, has a greater impact on MAM in pediatric UC.

*Phascolarctobacterium* was identified as the genus that was most differentially increased (moderate vs. inactive) or second most differentially increased (mild vs. inactive and severe vs. inactive) in MAM compared with inactive disease (Figure S5). This bacterial group is known to consume succinate [50,51,52], which might explain the increase in the succinate concentration at the mucous layer with mucosal inflammation. Succinate has been reported to act as a danger signal that induces macrophages to produce IL-1β [53]. Among the gut microbes, *Bacteroides*, *Parabacteroides*, and *Prevotella* produce succinate as an end product of sugar metabolism. The alteration of succinate metabolism in MAM with disease severity might be involved in mucosal inflammation in pediatric UC.

Finally, we identified potential microbial markers in MAM to discriminate UC from non-IBD pediatric patients (Figure S6). *Lactobacillus* and *Enterococcus* were identified as potential markers for UC, and others, including typical luminal bacteria such as butyrate-producing bacteria (*Faecalibacterium* and *Blautia*), were identified as potential markers for non-IBD pediatric patients. *Fusobacterium* of oral origin has been associated with periodontitis [54,55,56,57] and colon adenocarcinoma [58,59,60,61,62,63,64,65]. However, our results might indicate that the resident gut *Fusobacterium* plays an important role in keeping homeostasis in host-microbe interaction.

There are some limitations in this study. First, the small cohort size in this study was insufficient to reach statistical power in some analyses. Second, therapeutics may have affected the MAM, but therapeutic information for individual patients was not included in the analysis. The large-scale study cohort of pediatric UC is needed to adjust the clinical condition and medical treatment for reliable statistical analysis. Third, non-IBD controls in this study had abdominal symptoms, and healthy children were not enrolled as a control. Fourth, we could not do quantitative analyses (microbial cell density or short chain fatty acid level) due to the difficulty in adjusting the sampling volume. Finally, we did not perform the longitudinal evaluations in all the patients. Since UC is a condition with repeats in relapse and remission, we are now evaluating the longitudinal progress of MAM in individual patients. In addition, we need to analyze biopsy samples from pediatric UC and healthy controls in future studies to verify our hypothesis (compromised mucous layer function in UC), since biopsy samples contain the microbiota residing in the closest proximity to gut epithelial cells.

## 4. Materials and Methods

### 4.1. Patient Selection

This study was a single-center, prospective case series. All patients were enrolled from Kagawa University Hospital inpatient wards and outpatient pediatric clinics within the period 2020–2021. To participate in the study, patients had to have undergone colonoscopy for UC, CD, or suspected IBD; be willing to participate; and be able to maintain close follow-up. This study was approved by the Ethics Committee of Kagawa University (approval no: 2020-175). All patients and families gave written informed consent and assent in accordance with the Declaration of Helsinki. Clinical data of each participant are summarized in Table S1. The detailed information for patient cohort and clinical data used in this study has been described in our previous study [66]. All microbiome data was obtained in this study.

### 4.2. Colon Lavage Collection

Lavage samples were obtained from four sites (ascending colon, transverse colon, sigmoid colon, and rectum) by colonoscopy at active or remission phases of UC, or at the first colonoscopy in the cases for suspected IBD. Before the examination, patients ingested sodium picosulfate hydrate for colon cleansing the day before colonoscopy, and a bowel-cleansing agent was given orally on the day of the examination. If the patient could not take these agents or the patient’s bowel was not clear or fecal matter remained, an additional enema was performed. These drugs were used according to the age and acceptability of the patient. During colonoscopy, we selected the sites without luminal fluids or removed the fluids by aspiration. The target colonic mucosa was washed with 50 mL of saline, and the 20 mL of lavage samples were aspirated via a sterile tube and sampled using the working channel of the colonoscope. The aspiration tube was replaced at every collection site with a fresh one to avoid contamination. Each fluid sample was submitted to the study coordinator at room temperature and processed within 24 h after collection. We also collected stool samples 4 to 24 h before colonoscopy when available.

### 4.3. DNA Preparation

Colon lavage and fecal samples (0.1 g/mL in 1×PBS) were centrifuged at 12,000× *g* for 5 min to collect the bacterial cells, and the pellets were washed with 1×PBS (pH7.4). The suspensions were centrifuged again, and the pellets were resuspended in Tris-EDTA buffer (pH8.0). For 16S rRNA gene sequencing, DNA samples were extracted using the NucleoSpin DNA Stool kit (MACHEREY-NAGEL, Düren, Germany) according to the manufacturer’s instructions. DNA concentrations were determined using a Qubit dsDNA BR (Broad-Range) Assay kit (Thermo Fisher Scientific Inc., Waltham, MA, USA).

### 4.4. 16S rRNA Gene Analysis

Sequencing libraries were prepared by amplifying the V3-V4 hypervariable region of the 16S rRNA gene using the primer sets of 341F and 805R with Illumina Miseq adaptor sequence as previously reported [67]. Libraries were sequenced on the Illumina MiSeq platform using 300 bp paired-end V3 chemistry (Illumina Inc., San Diego, CA, USA) with the addition of 5% PhiX. Operational taxonomic unit (OTU) clustering of the sequencing reads was performed with Qiime2 with the UCLUST algorithm at 97% similarity against the Greengenes reference database (v.13.8). Sequence data were deposited to DDBJ. Correspondence between sample name and sequence data is listed in Table S2. Detailed sequencing statistics for each sample are summarized in Table S3.

### 4.5. Statistical Analysis

Statistical analyses were performed using the phyloseq package (v.1.50.0) in R (v.4.4.2). Calculation of alpha-diversity indices was performed using the MicrobiotaProcess package (v.1.18.0) [68]. Beta-diversity (Bray–Curtis distances) among samples was calculated with the vegan R package (v.2.6-10) based on the relative abundances of observed OTUs. Clustering of the samples was visualized by performing a principal coordinates analysis (PCoA) using the Bray–Curtis distance metric. A permutational multivariate analysis of variance (PERMANOVA) based on Bray–Curtis distances was performed using the adonis function of vegan with 9999 permutations. To account for the effect of repeated sampling from the same patient (CLFs and feces), differences in α-diversity were statistically examined by linear mixed-effects models, while differences in β-diversity were examined by repeated measures PERMANOVA. The monotonic relationship between clinical indices (Matts score or disease severity) and intra-individual Bray–Curtis dissimilarity was assessed using Spearman’s rank correlation analysis. The association between clinical metadata and bacterial taxa was analyzed using the MaAsLin2 package (v.1.20.0), where the calculated “coefficient value” represents the strength of the association. Differential analyses of OTU abundances were performed with the DESeq2 package (v.1.46.0) and linear discriminant analysis effect size (LEfSe) implemented in the MicrobiotaProcess package. For MaAsLin2, DESeq2, and other analyses involving multiple comparisons, *p*-values were adjusted to the False Discovery Rate (FDR) using the Benjamini–Hochberg method to control false positives. An FDR-adjusted *p*-value < 0.05 was considered statistically significant. Differences between two groups were tested using the Wilcoxon rank-sum test (Mann–Whitney *U*-test), and paired differences were determined using the Wilcoxon signed-rank test. Receiver operating curve (ROC) analysis was performed with the pROC package (v.1.19.0.1).

## 5. Conclusions

We herein analyzed a MAM in pediatric UC patients using CLFs. To the best of our knowledge, this is the first report to investigate the association of MAM and clinical data in pediatric UC patients. Our findings obtained in this study are as follows:

(i) The compositions of MAM were similar to fecal microbiota in pediatric UC patients, while they were significantly different in non-IBD, which might have resulted from the dysfunction of the mucous layer to separate MAM and luminal microbiota.
(ii) MAM in non-IBD showed higher α-diversity compared with feces, while only Enterobacteriaceae was enriched in MAM in pediatric UC.
(iii) Aggravation in UC disease severity or mucosal inflammation promotes inter-regional difference among the colonic MAMs, leading to the enrichment in microbes that usually reside in the oral cavity, oropharynx, upper intestine, or that have environmental origins.
(iv) Specific microbes in MAMs may be associated with inflammatory markers and humoral immune response, including autoantibodies.
(v) We identified nine potential microbial markers to discriminate pediatric UC from non-IBD patients.

We believe that these results provide novel insights into the association of mucosal bacterial communities with the pathophysiology of pediatric UC, in which severe pancolitis is prevalent. Analysis of the intestinal lavage samples from pediatric UC patients enables us to evaluate the microbiota residing proximal to the gut mucosa. In addition, the method is non-invasive, inexpensive, and suitable for longitudinal study. We are now planning such a longitudinal study with patients with pediatric UC to validate the microbial markers identified in this study for predicting prognosis.

## Figures and Tables

**Table 1:** Characteristics of enrolled pediatric UC and non-IBD patients.

	UC	Non-IBD
No. patient	19	19
%Male	52.6	57.9
Age (years) *	12.7 ± 3.1	10.1 ± 3.6
Disease Activity		
Active	10	−
Remission	9	−
Disease type		
Proctitis	2	−
Left-sided colitis	0	−
Extensive colitis	0	−
Pancolitis	17	−
No. CLF sample		
Ascending colon	18	19
Transverse colon	17	15
Sigmoid colon	19	18
Rectum	18	16
No. fecal sample	14	9
Matts score		
1	23	−
2	34	−
3−4	15	−
Stool test		
%FOB	36.0	30.6
Calprotectin (μg/g) **	1515 (376.5−3997.5)	49 (13.4−230.8)
Therapies		
Immunosuppressants	10	0
Biologics	7	0
Antibiotics	4	1

Among the non-IBD group, 6 patients had irritable bowel syndrome, 4 had functional abdominal pain syndrome, 4 had eosinophilic gastroenteritis, 3 had juvenile polyps, and 2 had constipation ± hemorrhoids. UC disease activity was scored by the Pediatric Inflammatory Ulcerative Colitis Index (active ≥ 10, remission < 10). UC disease type was categorized by Paris classification. Endoscopic findings of UC were categorized according to Matts classification (grade 1: normal or inactive, grade 2: mild, grade 3: moderate, and grade 4: severe). Antibiotics had been administered within 4 weeks before the examination. Abbreviations: CLF, colon lavage fluids. FOB: fecal occult blood. IBD: inflammatory bowel disease. UC: ulcerative colitis. * means ± standard deviations; ** median (interquantile range).

## Data Availability

The 16S rRNA gene sequencing read data have been deposited into DDBJ (Accession number: DRR609976-DRR610138).

## Supplementary Materials

The following supporting information can be downloaded at: https://www.mdpi.com/article/10.3390/ijms262110775/s1.

## Abbreviations

The following abbreviations are used in this manuscript:

Alb	Albumin
CLF	Colonic lavage fluid
cANCA	Cytoplasmic antineutrophil cytoplasmic antibodies
CD	Crohn’s disease
CRP	C-reactive protein
ESR	Erythrocyte sedimentation rate
F	Feces
FDR	False discovery rate
Fib	Fibrinogen
FOB	Fecal occult blood
Hct	Hematocrit
Hb	Hemoglobin
IBD	Inflammatory bowel disease
LEfSe	Linear discriminant analysis effect size
LDA	Linear discriminant analysis
MaAsLin2	Association analysis by linear model coefficients 2
MAM	Mucosa-associated microbiota
OTU	Operational taxonomic unit
pANCA	Peripheral antineutrophil cytoplasmic antibodies
PCoA	Principal coordinate analysis
PERMANOVA	Permutational multivariate analysis of variance
Plt	Platelet count
PUCAI	Pediatric Ulcerative Colitis Activity Index
ROC	Receiver operating characteristic
RM-PERMANOVA	Repeated measures PERMANOVA
SAA	Serum amyloid A protein
UC	Ulcerative colitis
WBC	White blood cell count
