# Supplementary material for: Comparative Analysis of Mucosa-Associated and Luminal Gut Microbiota in Pediatric Ulcerative Colitis
Source: Int J Mol Sci. 2025 Nov 5;26(21):10775. doi: 10.3390/ijms262110775 (PMC12610624; doi:10.3390/ijms262110775)
Supplement: Supplementary file 1 [file ijms-26-10775-s001.zip › Fig. S1_final.pdf]

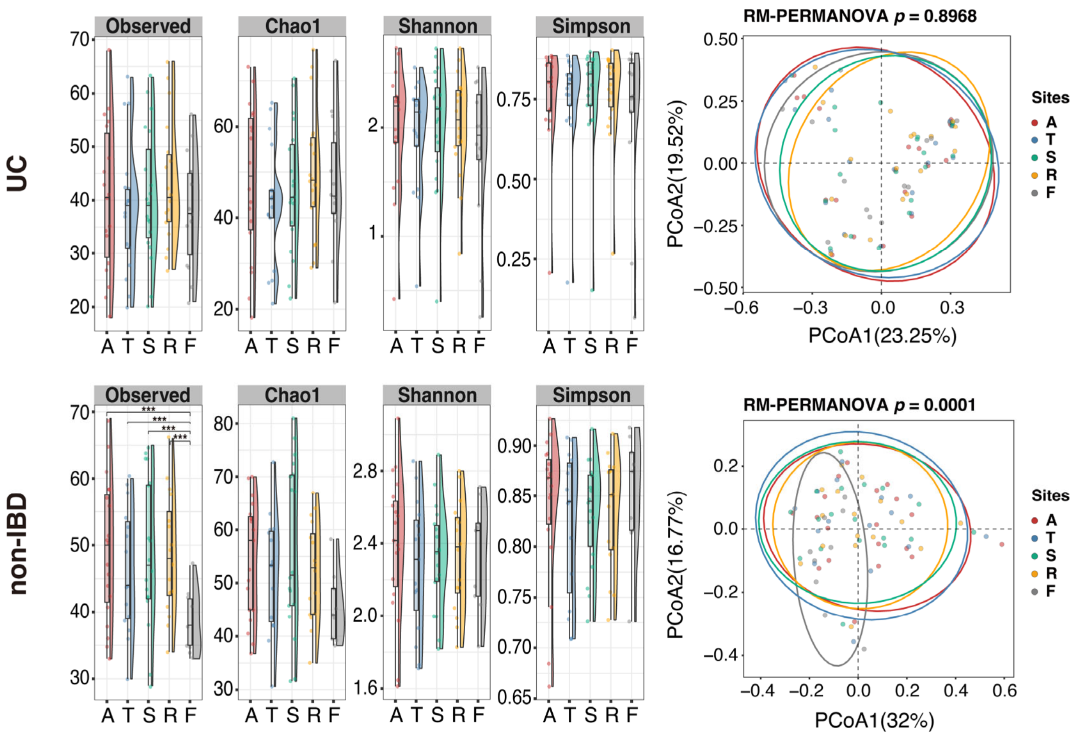

Figure S1. Comparisons of gut microbiome community structure among the sample sites in pediatric UC and non-IBD patients. The α-diversity (four kinds of diversity indices) and β-diversity (PCoA plot) of the microbiome were compared among sampling sites using a linear mixed-effects model and repeated measures PERMANOVA (RM-PERMANOVA; 9999 permutations), respectively. The circles indicate 95% confidence ranges for each sample, respectively. Upper panels (UC patients): No significant differences were observed among sampling sites in either α-diversity or in overall β-diversity (RM-PERMANOVA, $p = 0.8968$). Lower panels (non-IBD patients): For α-diversity, the number of observed number of genera was significantly higher in mucosa-associated samples (A, T, S, R) compared to feces (F). Asterisks indicate significant differences (*** $p < 0.001$). For β-diversity, the overall community structure also differed significantly among the sites (RM-PERMANOVA, $p = 0.0001$). Abbreviations: A, ascending colon; T, transverse colon; S, sigmoid colon; R, rectum; F, feces; IBD, inflammatory bowel disease; MAM, mucosa-associated microbiome; PCoA, principal coordinate analysis; UC, ulcerative colitis.
