# Supplementary material for: Comparative Analysis of Mucosa-Associated and Luminal Gut Microbiota in Pediatric Ulcerative Colitis
Source: Int J Mol Sci. 2025 Nov 5;26(21):10775. doi: 10.3390/ijms262110775 (PMC12610624; doi:10.3390/ijms262110775)
Supplement: Supplementary file 1 [file ijms-26-10775-s001.zip › Fig. S2_final.pdf]

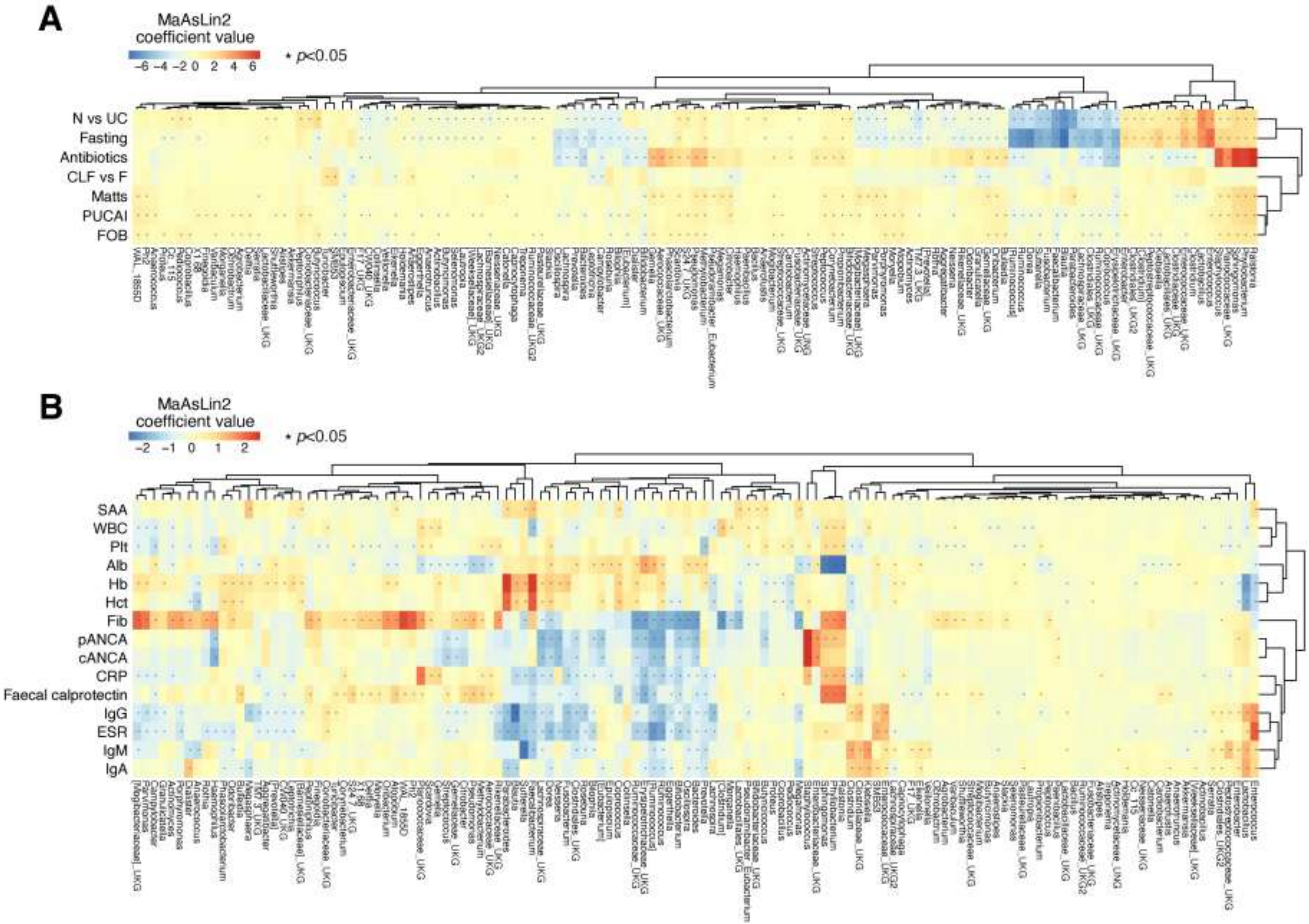
A
MaAsLin2
coefficient value
−6 −4 −2 0 2 4 6
* p<0.05
N vs UC
Fasting
Antibiotics
CLF vs F
Matts
PUCAI
FOB
B
MaAsLin2
coefficient value
−2 −1 0 1 2
* p<0.05
SAA
WBC
Plt
Alb
Hb
Hct
Fib
pANCA
cANCA
CRP
Faecal calprotectin
IgG
ESR
IgM
IgA

Figure S2. Association of the bacterial taxa (genus level) detected in CLF and feces with clinical metadata. Association was evaluated by linear model coefficients (MaAsLin2). Association strength is shown by the coefficient value with color gradation (red, positive; blue, negative). An asterisk (*) indicates a statistically significant association, defined as an FDR-adjusted p-value < 0.05. (A) Association of each microbial taxon with disease type (non-IBD vs UC), sampling sites, sample types, disease severity (PUCAI), disease activity (remission or active), mucosal integrity (Matts), FOB, fecal calprotectin level or treatment in the pediatric patients. (B) Microbial taxonomic variation plotted against blood and serological analysis data of the pediatric patients. Details of the clinical data are summarized in Table S1. Abbreviations: Alb, albumin; cANCA, cytoplasmic antineutrophil cytoplasmic antibodies; CLF, colon lavage fluids; CRP, C-reactive protein; ESR, erythrocyte sedimentation rate; F, feces; Fib, fibrinogen; FOB, fecal occult blood; Hb, hemoglobin; Hct, hematocrit; IBD, inflammatory bowel disease; pANCA, peripheral antineutrophil cytoplasmic antibodies; Plt, platelet count; PUCAI, Pediatric Ulcerative Colitis Activity Index; SAA, serum amyloid A protein; UC, ulcerative colitis; WBC, white blood cell count; UKG, unknown genus.
