# Supplementary material for: Comparative Analysis of Mucosa-Associated and Luminal Gut Microbiota in Pediatric Ulcerative Colitis
Source: Int J Mol Sci. 2025 Nov 5;26(21):10775. doi: 10.3390/ijms262110775 (PMC12610624; doi:10.3390/ijms262110775)
Supplement: Supplementary file 1 [file ijms-26-10775-s001.zip › Fig. S3_final.pdf]

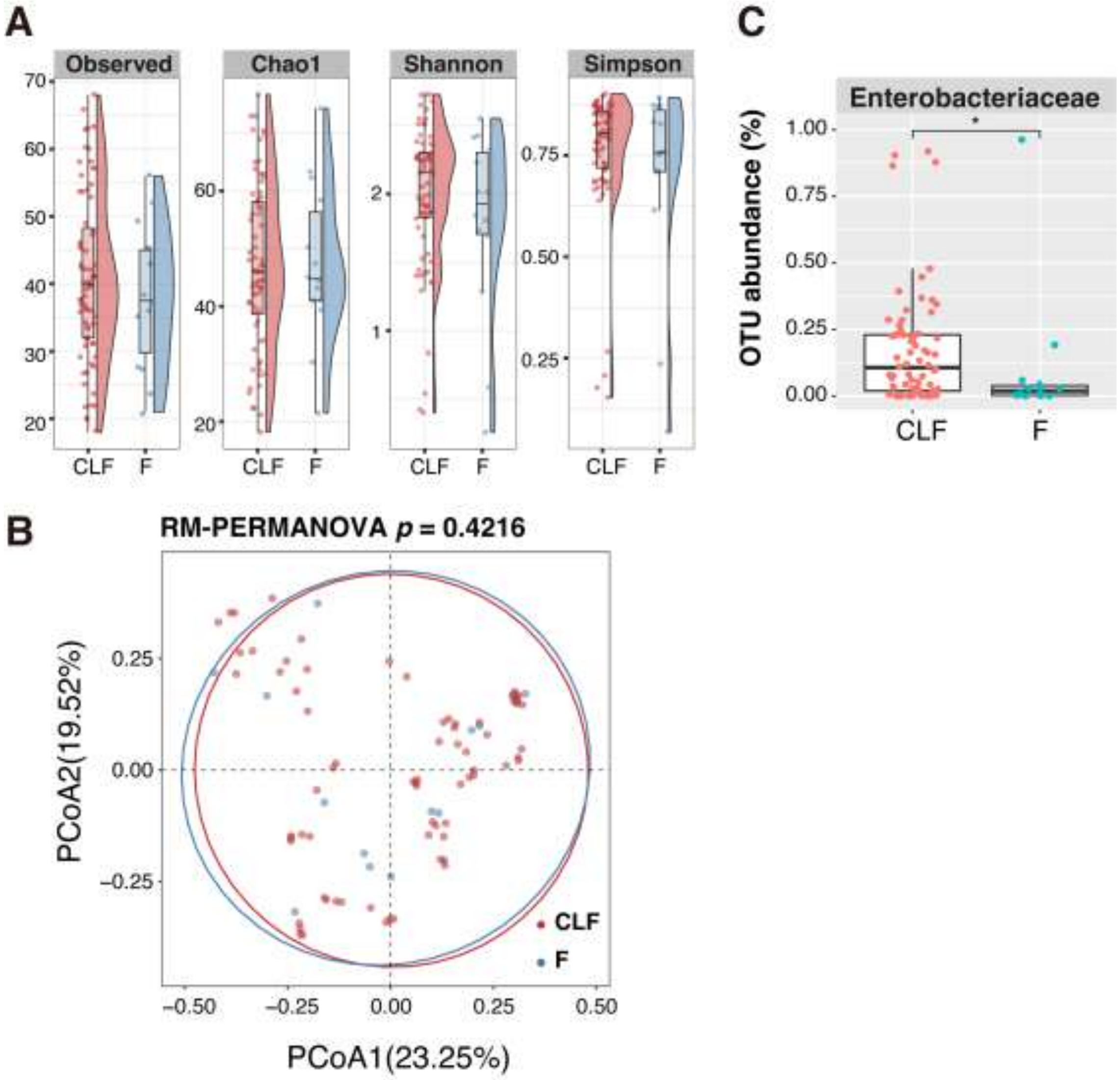

Figure S3. Difference between microbiota compositions of CLF and feces in pediatric UC patients. (A) Comparison of α-diversity indices between the gut microbiome in CLF and F samples. No significant differences were observed (linear mixed-effects model). (B) PCoA of the gut microbiome based on Bray-Curtis dissimilarity. The overall community structure did not differ significantly between CLF and F ($p = 0.4216$; RM-PERMANOVA, 9999 permutations). The circles indicate 95% confidence ranges for each sample type, respectively. (C) Relative abundance of Enterobacteriaceae, which was the only bacterial family found to be significantly different between CLF and F. The *y*-axis represents the OTU abundance (%). The asterisk indicates a significant difference ($p < 0.05$; Wilcoxon signed-rank test). Abbreviations: CLF, colon lavage fluids; F, feces; PCoA, principal coordinate analysis; RM-PERMANOVA, repeated measures PERMANOVA.
