# Supplementary material for: Comparative Analysis of Mucosa-Associated and Luminal Gut Microbiota in Pediatric Ulcerative Colitis
Source: Int J Mol Sci. 2025 Nov 5;26(21):10775. doi: 10.3390/ijms262110775 (PMC12610624; doi:10.3390/ijms262110775)
Supplement: Supplementary file 1 [file ijms-26-10775-s001.zip › Fig. S4_final.pdf]

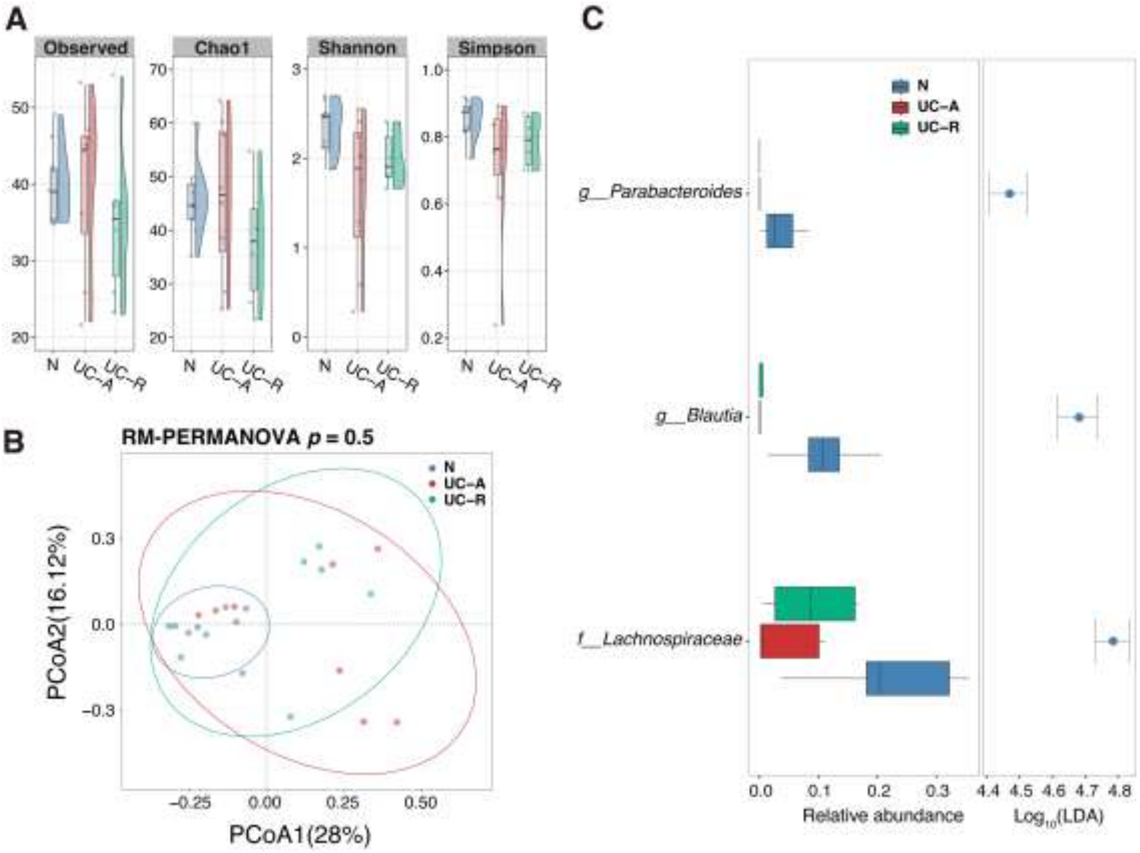

Figure S4. Difference between microbial community structures in feces between pediatric UC and non-IBD patients. (A) Comparison of α-diversity indices in feces among the non-IBD (N), active UC (UC-A) and remission UC (UC-R) groups. No significant differences were observed among the groups. (B) PCoA plot based on Bray-Curtis dissimilarity. The overall community structure in feces did not differ significantly among the N, UC-A, and UC-R groups ($p = 0.5$; RM-PERMANOVA, 9999 permutations). The circles indicate 95% confidence ranges for each group, respectively. (C) LEfSe analysis identifying taxa that are differentially abundant among the groups. The plot highlights taxa found to be enriched in the non-IBD (N) group. The left side shows the relative abundance of each taxon, while the right side shows its corresponding linear discriminant analysis (LDA) score. Abbreviations: LDA, linear discriminant analysis; N, non-IBD (non-inflammatory bowel disease); PCoA, principal coordinate analysis; RM-PERMANOVA, repeated measures PERMANOVA; UC-A, active ulcerative colitis; UC-R, remission ulcerative colitis.
