# Supplementary material for: Comparative Analysis of Mucosa-Associated and Luminal Gut Microbiota in Pediatric Ulcerative Colitis
Source: Int J Mol Sci. 2025 Nov 5;26(21):10775. doi: 10.3390/ijms262110775 (PMC12610624; doi:10.3390/ijms262110775)
Supplement: Supplementary file 1 [file ijms-26-10775-s001.zip › Fig. S5_final.pdf]

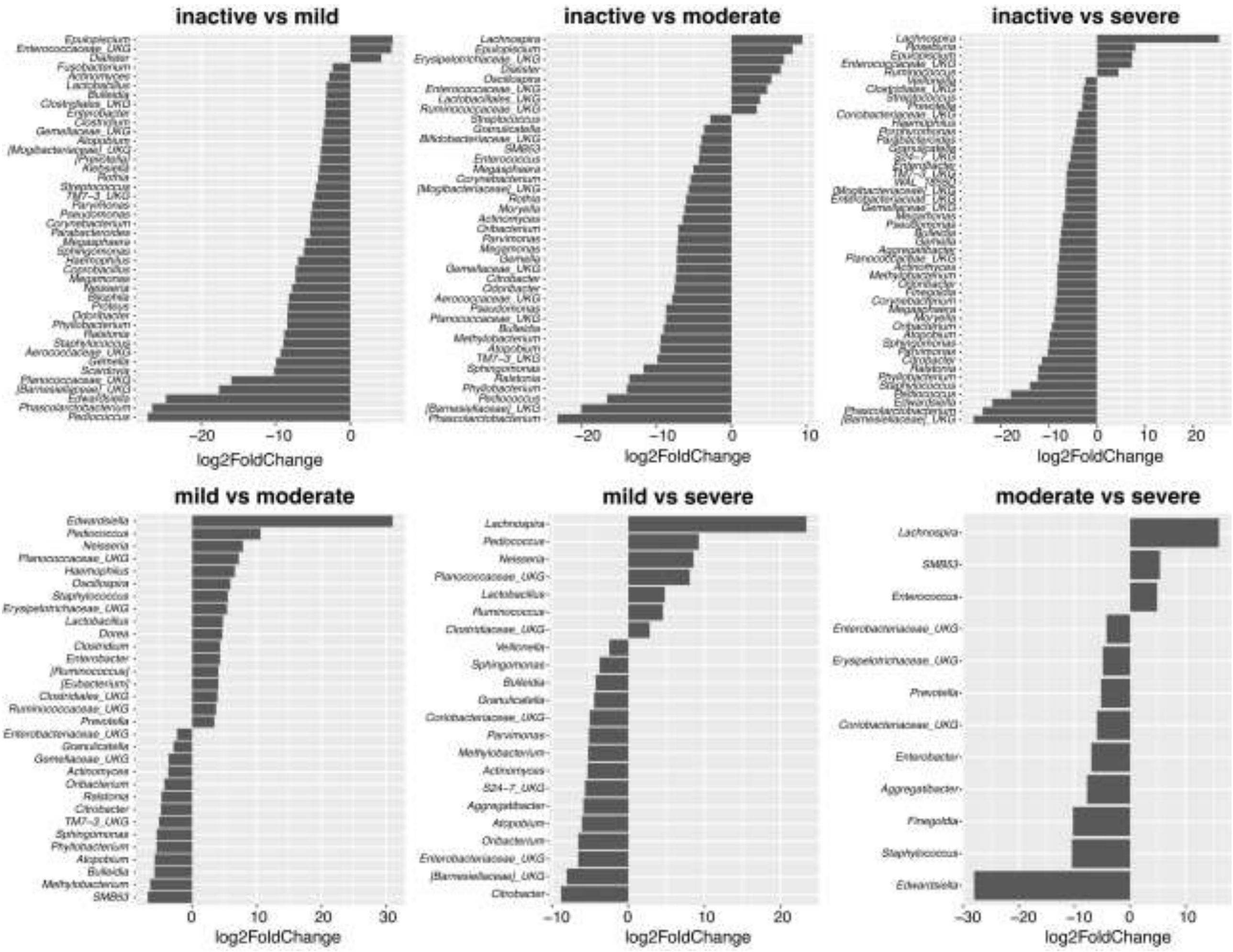

Figure S5. Differentially abundant bacterial genera in MAM identified by pairwise comparisons among different degrees of disease activity. DESeq2 analysis was used to identify bacterial genera that were differentially enriched among paired disease activities. The bar plots show the log2 fold change for each comparison. A positive value indicates enrichment in the former disease condition (e.g., in 'inactive' for the 'inactive vs mild' comparison), while a negative value indicates enrichment in the latter. Only genera with an FDR-adjusted *p*-value < 0.05 and a fold change greater than 4 are shown. Abbreviation: MAM, mucosa-associated microbiome.
