# Supplementary material for: Comparative Analysis of Mucosa-Associated and Luminal Gut Microbiota in Pediatric Ulcerative Colitis
Source: Int J Mol Sci. 2025 Nov 5;26(21):10775. doi: 10.3390/ijms262110775 (PMC12610624; doi:10.3390/ijms262110775)
Supplement: Supplementary file 1 [file ijms-26-10775-s001.zip › Fig. S6_final.pdf]

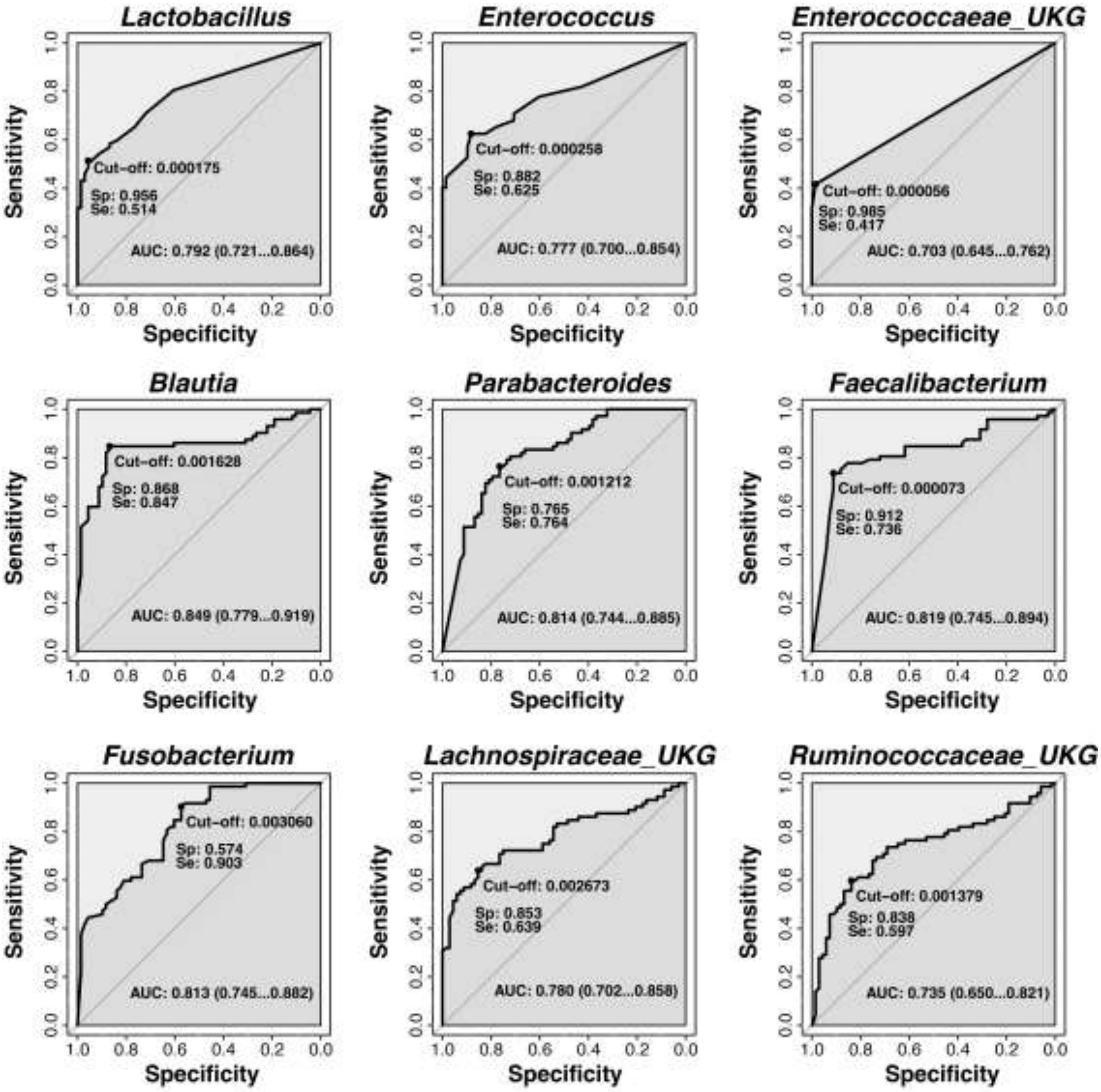

Figure S6. ROC analysis of selected bacterial taxa in MAM as potential diagnostic markers for pediatric UC. ROC curve analysis was performed on the relative abundance of nine selected bacterial taxa to evaluate their potential to discriminate between pediatric UC and non-IBD patients. Each plot shows the ROC curve, the Area Under the Curve (AUC) with its 95% confidence interval, and the optimal cut-off value with the corresponding sensitivity (Se) and specificity (Sp). Abbreviations: AUC, area under the curve; MAM, mucosa-associated microbiome; non-IBD, non-inflammatory bowel disease; ROC, receiver operating characteristics; Se, sensitivity; Sp, specificity; UC, ulcerative colitis.
