# Supplementary material for: Comparative Analysis of Mucosa-Associated and Luminal Gut Microbiota in Pediatric Ulcerative Colitis
Source: Int J Mol Sci. 2025 Nov 5;26(21):10775. doi: 10.3390/ijms262110775 (PMC12610624; doi:10.3390/ijms262110775)
Supplement: Supplementary file 1 [file ijms-26-10775-s001.zip › Fig. S7_final.pdf]

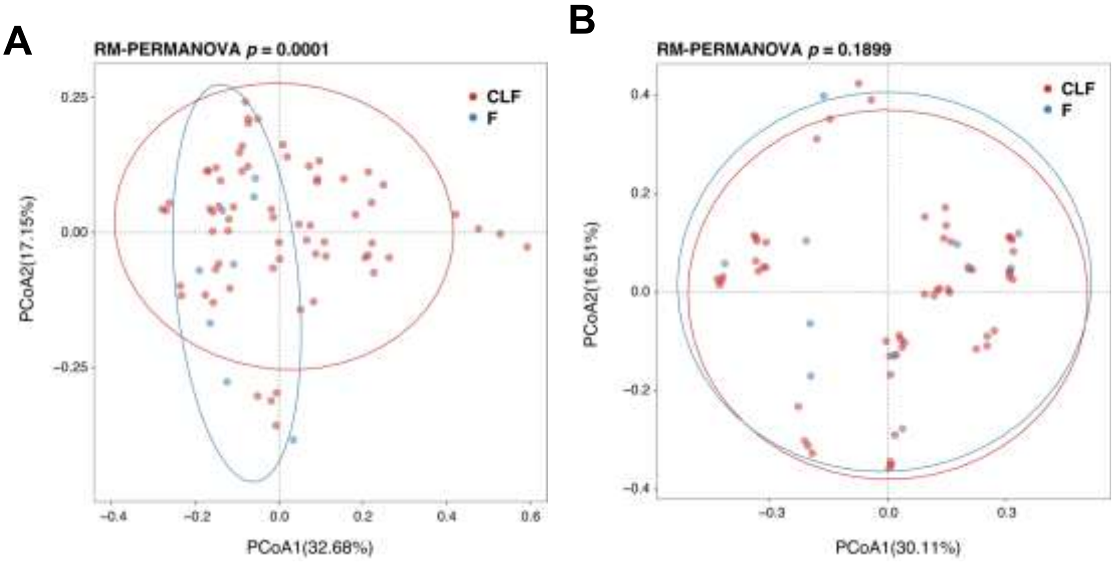

Figure S7. Difference in microbiota composition between CLF and feces in non-IBD (A) and UC patients(B) when the data from the patient with antibiotic treatment within 4 weeks before sampling were excluded. PCoA plot based on Bray–Curtis dissimilarity, showing that the microbial communities of CLF and F form statistically distinct clusters only in non-IBD group ($p = 0.0001$; RM-PERMANOVA, 9999 permutations). The circles indicate 95% confidence ranges for each sample type, respectively. The plot in panel A and B corresponds to Figure 3B and Figure S3B, respectively.
